# Exploring the Causal Pathway From Telomere Length to Alzheimer’s Disease: An Update Mendelian Randomization Study

**DOI:** 10.3389/fpsyt.2019.00843

**Published:** 2019-11-15

**Authors:** Kai Gao, Chen Wei, Jin Zhu, Xin Wang, Guoqing Chen, Yangyang Luo, Dai Zhang, Weihua Yue, Hao Yu

**Affiliations:** ^1^Peking-Tsinghua Center for Life Sciences, Academy for Advanced Interdisciplinary Studies, Peking University, Beijing, China; ^2^NHC Key Laboratory of Mental Health, National Clinical Research Center for Mental Disorders, Ministry of Health, Peking University Sixth Hospital (Institute of Mental Health), Peking University, Beijing, China; ^3^Department of Psychiatry, Jining Psychiatric Hospital, Jining, China; ^4^Department of Psychiatry, Jining Medical University, Jining, China; ^5^PKU-IDG/McGovern Institute for Brain Research, Peking University, Beijing, China

**Keywords:** telomere length, Alzheimer’s disease, genome-wide association study, genetic instrument, Mendelian randomization

## Abstract

Increasing evidence shows that telomere length shortening is associated with the risk for Alzheimer’s disease (AD), pointing to a potential modifiable target for prevention. However, the causality of this association is still not clear. To investigate the causal relationship between telomere length and AD, we use two-sample Mendelian randomization (MR) to assess potential causal inference. We used summary-level data for telomere length (9,190 participants) and AD (71,880 cases and 383,378 controls). We performed two-sample MR analysis with single nucleotide polymorphisms previously identified to be associated with telomere length. The MR analyses were conducted using the inverse-variance-weighted method and complemented with the maximum likelihood, weighted median, weighted mode approaches. MR evidence suggested that shorter telomere length was causally associated with a higher risk for AD (inverse-variance weighted estimate of odds ratio (OR): 1.03 per SD decrease of telomere length, *P*=1.21×10^−2^). The maximum likelihood, weighted median, weighted mode yielded a similar pattern of effects. The results were similar in sensitivity analyses. Using genetic instruments identified from large-scale genome-wide association study, robust evidence supports a causal role of telomere length shortening with increased risk of AD.

## Introduction

Alzheimer’s disease (AD) is the most common neurodegenerative disorder characterized by cognitive and behavioral impairment, social and occupational dysfunction and, ultimately, death. Advancing age is a major risk factor for AD, both the prevalence and global burden of AD increase with age, especially between the ages of 50 and 80 years (1). AD, accounting for 50–70% of all dementia cases, is the main cause of dementia, which is the fifth leading cause of death worldwide (1,). The aetiology of AD is not well understood, it is well recognized that both environment and genetic factors are contribute to the development of AD (3). Telomeres, capping the ends of chromosomes, are DNA–protein complexes consist of repetitive nucleotide sequences (TTAGGG) repeats protecting the DNA from damage and are important for chromosomal stability and cellular integrity (4). Telomeres are shorten progressively over time during each cell division, thus, they are recognized as physiological markers of aging (5). Alterations of telomere length are proposed as epigenomic markers associated with a wide range of diseases, including cancer, cardiovascular diseases, neurological disorders, and psychiatric diseases (6–10).

Meta-analysis studies have indicated that shorter telomeres is associated with AD (11, 12), telomere shortening may be an indicator of AD progression (13, 14). However, longitudinal studies did not find the association between shorter telomeres and AD (15). Telomere shortening was reported to slow down the progression of AD in mouse model (16). In addition, AD was reported to accelerate telomere loss (17, 18), indicating AD might be a risk factor for telomere shortening. As a result, the findings are inconsistent or even contradictory, and it is difficult to make sure the causality between telomere length shortening and AD because of residual confounding or reverse causation.

The aim of the present study was to perform a Mendelian randomization (MR) study, using germline genetic variants as proxies for telomere length, to test the causality between an exposure (telomere length) and an outcome (AD) (19). Because alleles inherited from both parents are randomly distributed to offspring, and single nucleotide polymorphisms (SNPs) associated with telomere length used as instrumental variables randomly distributed throughout an unbiased general population, the approach can estimate the causality association of telomere length and risk of AD. Two MR studies have suggested that SNPs associated with telomerase length shortening as causative for AD (20, 21), however, the selected number of risk SNPs for telomerase length is only seven. Herein, we leverage summary genetic associations from genome-wide association study (GWAS) data of telomerase length and AD to assess the causal relationship of telomerase length with the risk for AD.

## Methods

### Genetic Instrumental Variables

To select genetic instrumental variables, we used 16 SNPs (*P* < 5×10^−8^) previously identified to be genome-wide associated with telomere length, which were curated by the GWAS catalog on January 15, 2015 (9). We also searched the GWAS catalog on July 23, 2019, and found that there was no update of telomere length GWAS in European population (22). The summary data for all 16 SNPs (i.e., allele frequency, beta value, standard error, and *P* values) were acquired from a meta-analysis of GWASs of telomere length, involving 9,190 participants of European ancestry (23). This method for selecting genetic instruments has been used in the previous MR research when more potential instruments are needed (9).

### Alzheimer’s Disease Genome-Wide Association Study Data

We used summary-level data from a recent large-scale AD GWAS (24). To reduce potential bias from population stratification, we only drew on summary-level data for AD from European-descent individuals, consisting of 71,880 AD cases and 383,378 controls (24). The cases were clinically diagnosed or by-proxy. The AD case diagnosed by proxy status is based on parental AD diagnosis. Previous study showed that AD-by-proxy was significantly correlated with clinically diagnosed AD (24). Summary data were downloaded from the website (https://ctg.cncr.nl/software/summary_statistics). More details about sample description, genotyping, and statistical analyses can be found in the original paper (24).

### Statistical Analysis

**Figure 1:**
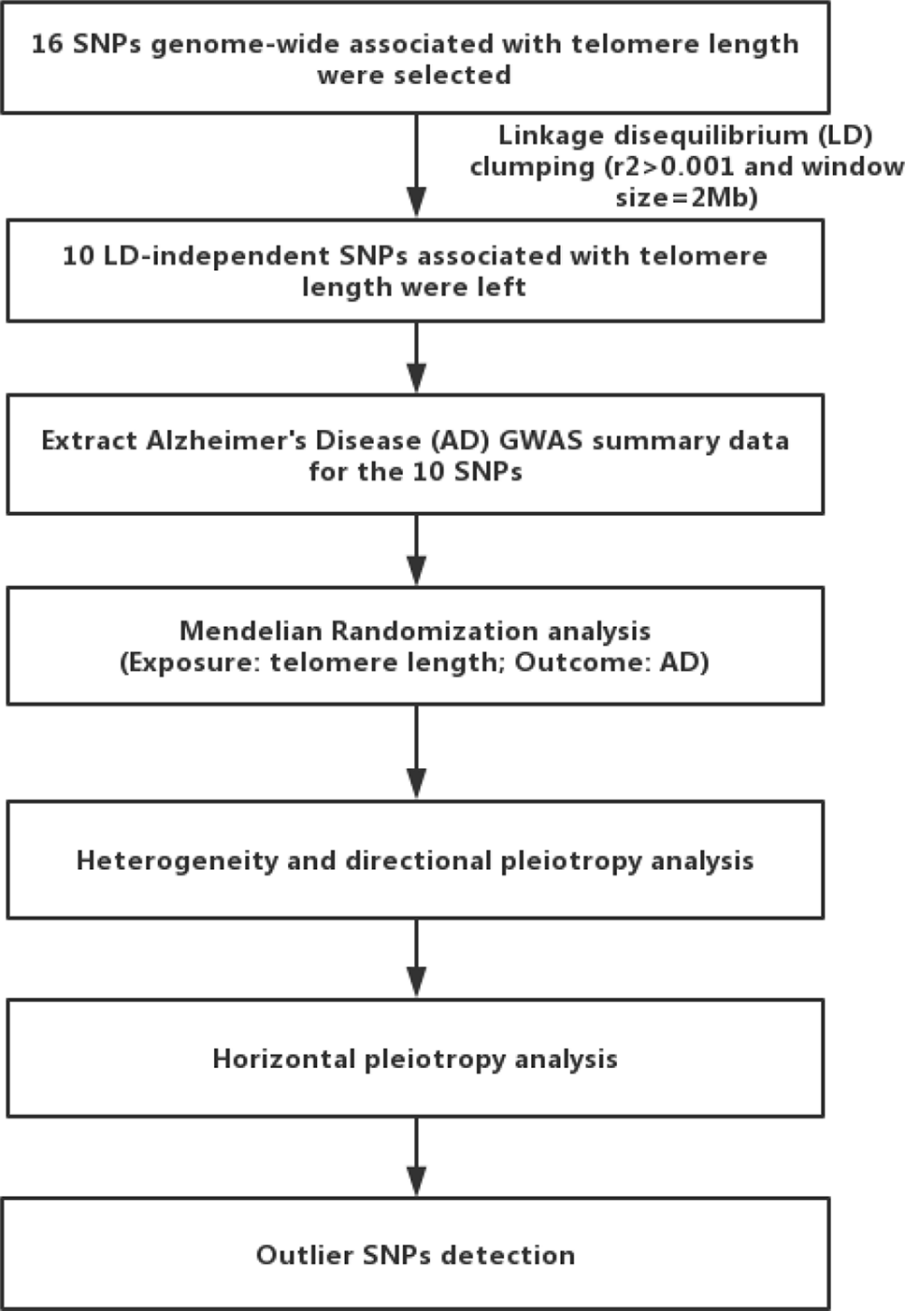
Flow chart of the present study. First, we selected 16 single nucleotide polymorphisms (SNPs) associated with telomere length as genetic instrumental variables. After linkage disequilibrium-based clumping (r^2^ 0.001 and window size = 2 Mb), 10 SNPs were used as instrumental variables. Then, we used the inverse-variance weighted method to combine SNP-specific causal estimates for AD, complementing with the maximum likelihood, weighted median, weighted mode approaches. Finally, we detected heterogeneity and directional pleiotropy of the genetic instruments, performed horizontal pleiotropy to detect heterogeneous outcomes, and detected outlier SNPs.

MR analyses were conducted in the R computing environment using the TwoSample MR package (25). The genome-wide significantly (*p* < 5×10^−8^) associated SNPs for telomere length or AD were selected. To selected the independent SNPs (r^2^ > 0.001 and window size = 2 Mb), we performed linkage disequilibrium (LD) clumping using PLINK v1.9 (26). The 1,000 Genomes Project Phase 3 European datasets were used to calculate LD between the variants. The inverse-variance weighted (IVW) method was adopted to combine SNP-specific causal estimates for AD (27), complementing with the maximum likelihood, weighted median, weighted mode approaches (25). We used a weighted median function (28) and MR-Egger regression (29) to detect heterogeneity and directional pleiotropy of the genetic instruments. To examine robustness of significant results, we performed horizontal pleiotropy through meta-analytic methods to detect heterogeneous outcomes, such as leave-one-SNP-out analyses, the modified Cochran Q statistic, and the MR Egger intercept test (25). To detect pleiotropy and outlier SNPs, we also used the MR-Pleiotropy RESidual Sum and Outlier (MR-PRESSO) (30). These tests could essentially capture the extent to which the effect for one or more instrument SNP is exaggerated in magnitude. All statistical analyses were performed using R (v 3.5.0) and the related packages (TwoSample MR and MR-PRESSO) (25, 30). Additionally, we also compared our MR results with a meta-analysis of traditional observational studies of telomere length in AD (11) using Cochran Q statistic. To explain the workflow of the study, a flow chart about the analytical methods and how the MR analysis were performed step-by-step was shown in **Figure 1**.

## Results

**Figure 2:**
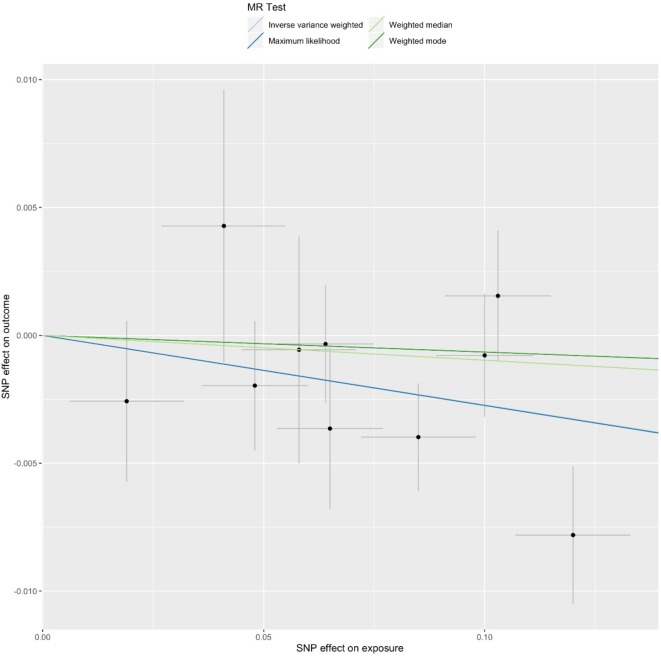
Mendelian randomization (MR) plots for relationship of telomere length with Alzheimer’s disease (AD). Analyses were conducted using the conventional inverse-variance-weighted MR method and complementary methods, including maximum likelihood, weighted median, weighted mode approaches. Scatterplot of SNP potential effects on telomere length with AD, with the slope of each line corresponding to estimated MR effect per method.

**Figure 3:**
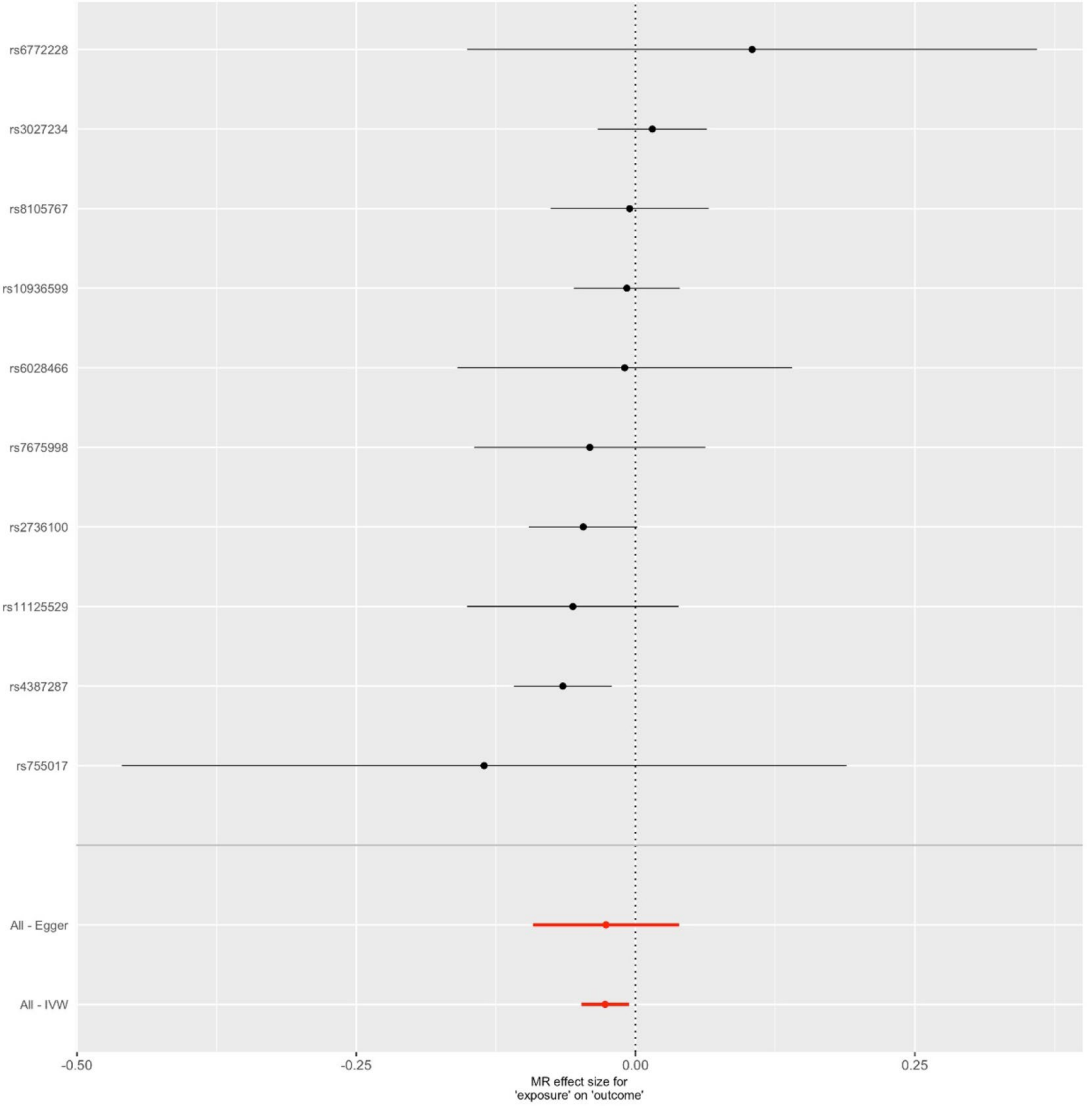
Forest plot for genetic and causal effects of telomere length on Alzheimer’s disease (AD). The effects of telomere length associated variants on AD using genome-wide association study data. The OR could be interpreted as changes in odds per telomere length decreasing allele for AD.

**Figure 4:**
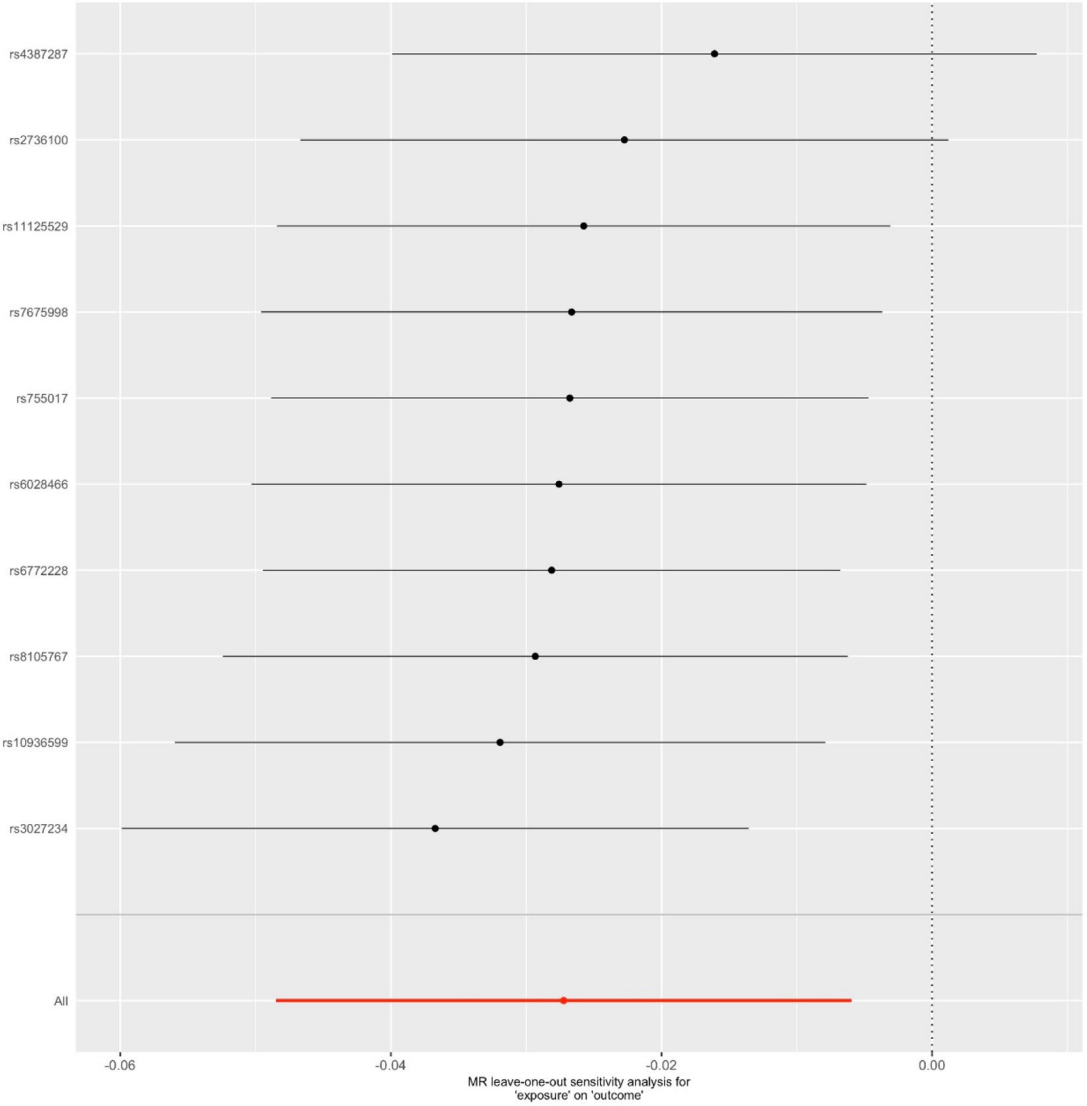
Leave-one-out sensitivity analysis for the final instrument variable set. The solid lines represent 95% confidence intervals.

We first selected 16 SNPs identified to be associated with telomere length in Europeans as instrumental variables (9, 23). After clumping (*r* *^2^* > 0.001 and window size = 2 Mb), 10 SNPs were used as instrumental variables for telomere length (**Supplementary Table 1**). Summary data for the genetic instruments were available for telomere length (**Table 1**). In MR analysis, we found evidence of a causal relationship between telomere length shortening and AD (IVW estimate of odds ratio (OR): 1.03 per 1-SD decrease in genetically determined telomere length, 95% confidence interval (CI): 1.01–1.05, *P* = 1.21×10^−2^; **Figure 2** and **Table 2**). The maximum likelihood, weighted median and weighted mode yielded a similar pattern of effects (**Figure 2** and **Table 2**). To investigate the consistency and directional effect of the SNP association with telomere length and AD, we plotted the effect and standard error of SNPs on telomere length with their corresponding effect, and standard error on the risk of AD for each data set (**Figure 3**). Furthermore, analyses leaving out each SNP revealed that no single SNP drove these results but rather reflected an overall combined pattern of opposite relationships with telomere length shortening and AD (**Figure 4**). Similarly, we observed no heterogeneity in the effect estimates for the 10 independent telomere length associated SNPs in Europeans (IVW: *P* = 0.41; MR Egger: *P* = 0.32). The MR-PRESSO test also showed outlier pleiotropy and suggested no SNP outliers (*P* = 0.29). Additionally, there was no evidence of directional pleiotropy in the MR-Egger analysis (*P* = 0.98). Furthermore, our MR results were generally similar in direction and magnitude to estimates based on observational prospective studies of telomere length and AD (heterogeneity test, *P* = 0.97; **Supplementary Figure 1**).

**Table 1:** The genetic instruments for Mendelian randomization analysis of telomere length (exposure) and Alzheimer’s disease (outcome).

SNP	Chr	BP	Gene	A1	A2	Freq_TL	Freq_AD	Beta_TL	SE_TL	*P*_TL	Beta_AD	SE_AD	*P*_AD	Removed^a^
rs10936599	3	169774313	TERC	C	T	0.76	0.75	1.00E−01	1.10E−02	3.00E−31	−7.83E−04	2.42E−03	7.46E−01	No
rs8105767	19	22032639	ZNF208	G	A	0.25	0.30	6.40E−02	1.10E−02	1.11E−09	−3.39E−04	2.31E−03	8.83E−01	No
rs11125529	2	54248729	ACYP2	A	C	0.16	0.13	6.50E−02	1.20E−02	8.00E−10	−3.65E−03	3.14E−03	2.46E−01	No
rs4387287	10	103918139	OBFC1	A	C	0.14	0.19	1.20E−01	1.30E−02	2.00E−11	−7.81E−03	2.69E−03	3.66E−03	No
rs2736100	5	1286401	TERT	C	A	0.52	0.50	8.50E−02	1.30E−02	4.38E−19	−3.98E−03	2.10E−03	5.85E−02	No
rs3027234	17	8232774	CTC1	C	T	0.83	0.79	1.03E−01	1.20E−02	2.00E−08	1.54E−03	2.57E−03	5.48E−01	No
rs755017	20	63790269	ZBTB46	G	A	0.17	0.13	1.90E−02	1.29E−02	6.71E−09	−2.58E−03	3.14E−03	4.13E−01	No
rs7675998	4	163086668	NAF1	G	A	0.80	0.78	4.80E−02	1.20E−02	4.35E−16	−1.97E−03	2.53E−03	4.38E−01	No
rs6028466	20	39500359	DHX35	A	G	0.17	0.06	5.80E−02	1.30E−02	2.57E−08	−5.65E−04	4.43E−03	8.98E−01	No
rs6772228	3	58390292	PXK	T	A	0.87	0.96	4.10E−02	1.40E−02	3.91E−10	4.28E−03	5.34E−03	4.23E−01	No
rs1317082	3	169779797	TERC	A	G	0.71	0.75	9.70E−02	1.10E−02	1.00E−08	−8.53E−04	2.42E−03	7.25E−01	Yes
rs412658	19	22176638	ZNF676	T	C	0.35	0.36	8.60E−02	1.00E−02	1.00E−08	−1.08E−03	2.19E−03	6.20E−01	Yes
rs12696304	3	169763483	TERC	C	G	0.74	0.71	9.00E−02	1.10E−02	4.00E−14	−6.68E−04	2.31E−03	7.73E−01	Yes
rs9419958	10	103916188	OBFC1	T	C	0.13	0.16	1.29E−01	1.30E−02	9.00E−11	−8.91E−03	2.91E−03	2.19E−03	Yes
rs9420907	10	103916707	OBFC1	C	A	0.14	0.15	1.42E−01	1.40E−02	7.00E−11	−9.18E−03	2.91E−03	1.59E−03	Yes
rs10936601	3	169810661	TERC	C	T	0.74	0.71	8.70E−02	1.10E−02	4.00E−15	−7.62E−04	2.32E−03	7.42E−01	Yes

SNP, single-nucleotide polymorphism; Chr, chromosome; BP, base position; A1/A2, reference allele/alternate allele; Freq_TL, the frequency of reference allele in TL GWAS; Freq_AD, the frequency of reference allele in AD GWAS; BETA_TL, SE_TL, and P_TL, association analysis with the telomere length; BETA_AD, SE_AD, and P_AD for association analysis with AD. ^a^SNPs removed due to LD.

**Table 2:** Mendelian randomization results for the relationship between telomere length and Alzheimer’s disease.

Method	OR (95% CI)^a^	*P*-value	No. of SNPs
Inverse variance-weighted	1.03 (1.01–1.05)	1.21E−02	10
Maximum likelihood	1.03 (1.01–1.05)	1.13E−03	10
Weighted mode	1.01 (0.96–1.05)	7.66E−01	10
Weighted median	1.01 (0.98–1.04)	5.25E−01	10

a Indicates odds for AD per 1-SD decrease in genetically determined risk of telomere length.

## Discussion

Using large-scale GWAS data for telomere length (N = 9,190) and AD data (71,880 AD cases and 383,378 controls), we performed MR analysis to assess the causal relationship between telomere length and AD using a two-sample MR analysis. With genetic variants as proxies for the telomere length, the MR analysis supports the evidence from conventional analyses that telomere length shortening is associated with increased risk of AD. Our study confirms and extends previous findings by applying complemented MR analyses and several sensitivity analyses.

### Comparison With Previous Studies

Our MR findings are generally consistent with those conventional observational studies, which tend to report associations of telomere length shortening with increased risk of AD. A multiethnic 9-year followed up study found that shortened leukocyte telomere length is associated with risk for AD, and the risk for AD increased 21% for each kilobase pair of decreased telomere length (31). Likewise, another 2-year follow-up study showed reduced telomere length significantly correlated with dementia in stroke patients (32). Longitudinal investigations did not support the association between shorter telomeres and AD (15, 33). However, the recent meta-analyses with large sample size provided evidence for shorter telomere length and increased risk of AD (11, 12), and most studies have found shorter telomere length in the leukocytes of AD patients (34). In our analysis, shorter telomere length, proxied by 16 genetic variants, was significantly associated with risk of AD.

### Underlying Mechanisms of Telomere Length Shortening in Alzheimer’s Disease

The genetic variants used as instrumental variables contain 11 genes, most of which were reported to be involved in telomere biology (35), and some of them may play a role in the pathophysiology of AD (21, 36). Telomerase RNA component (*TERC*) gene, encoding one of the main components of telomerase, provides as a template for addition of multiple “TTAGGG” repeats. While telomerase reverse transcriptase (*TERT*) gene, encoding the catalytic subunit of telomerase (37). SNPs in the two genes shown in the **Table 1** were all significantly associated with telomere length and carriers of the reference alleles of these SNPs had significantly longer telomeres, and these common variants were reported to be implicated in AD susceptibility (38). The oligonucleotide/oligosaccharide-binding folds containing one (*OBFC1*) gene encodes a protein specifically participating in the replication and capping of telomeres (39). The gene telomere maintenance complex component 1 (*CTC1*) maintains telomere and is required for telomere integrity (40). NAF1, one of subunit of the tetrameric complex of dyskerin, which is an essential component of the telomerase enzyme (41). The *ZNF676*, *ZBTB46,* and *ZNF208* genes encode zinc finger proteins directly binding to DNA. They may modify telomere length through altering the expression of genes engaged in telomere maintenance or inhibiting telomere elongation by binding specifically to G-quadruplex at the 3’ end of the telomeres (23). However, how *ACYP2*, *DHX35,* and *PXK* genes are implicated in telomere length regulating is not clear, but it has reported the family member of DHX35 is required for telomere protection (42). All the SNPs are significantly associated with telomere length (*P* < 5 × 10^−8^), and the reference alleles of these SNPs in the 11 genes are significantly related with longer telomeres (**Table 1**). Moreover, few other zinc finger proteins have been found to contribute to the pathogenesis of AD (43, 44). *ACYP2*, encoding an acylphosphatase regulating Ca^2+^ homeostasis, dysregulation of which is a key step in the pathogenesis of AD (45, 46).

### Clinical Relevance of Findings

Our findings support a causal link between telomere attrition and AD, providing potential clinical applications. First, as telomere length is largely inherited (47), individuals who inherit shorter telomere length may be predisposed to AD, making leukocyte telomere length an appealing candidate for AD prediction. Second, factors affecting telomere length at birth, such as sex (48), paternal age at conception (49), environmental factors during gestation (50), will have substantial health impact on postnatal life, suggesting telomere length may be the mediator of *in utero* exposures on the onset of AD later in life. Third, telomere length may provide as an intervention target for AD prevention, because shorter telomere length is widely accepted as an indicator of poorer health status (4), measurement of telomere length services to the public to motivate healthy lifestyle choices in individuals.

### Study Limitations

There were also some limitations in our study. First, our results might be confounded by pleiotropy, population stratification. The majority of our results are consistent in sensitivity analyses that made allowance for violations of MR assumptions. Population stratification was reduced in our study because both telomere length and AD GWAS were restricted to individuals of European ancestry. Second, the AD GWAS studies had the limitation that the diagnosis of AD was partly based on proxy. The AD case diagnosed by proxy status is based on parental AD diagnosis, however, AD-by-proxy has been demonstrated to be predictive of AD diagnosis. Previous study showed that AD-by-proxy was significantly correlated with clinically diagnosed AD (24). Third, the telomere length from the GWAS was from leukocyte, but not the brains of AD patients. Considering brain samples are not easily accessible and there is little cell turnover between leukocyte telomere length and other tissues (51). Telomere length was reported to reduce with the rate of 29–60 bp/year in most tissues except cerebral cortex, owing to the limited cell proliferation in brain (52). Very few studies have examined telomere length in brain tissues of AD patients, and the associations between telomere length in different brain tissues and AD are contradictory (53, 54), but leukocyte telomere length was significantly correlated with both brain telomere length and structural brain changes (54–56), as a result, it is proposed that telomere length in leukocyte can be a proxy of neuronal telomere length.

In summary, our findings provide evidence in support of the causal role of telomere length shortening in the risk of AD.

## Data Availability Statement

The datasets generated for this study can be found in the doi: 10.1001/jamaoncol.2016.5945., https://ctg.cncr.nl/software/summary_statistics.

## Ethics Statement

Written informed consent was obtained from the individual(s) for the publication of any potentially identifiable images or data included in this article.

## Author Contributions

KG, HY, and WY designed the study. KG, HY, and WY contributed to analysis and interpretation of data, and wrote the first draft of the manuscript. KG, XW, JZ, GC, YL, and HY did the statistical analyses and prepared the tables and figures. WC, DZ, and WY revised the manuscript. All authors contributed to drafting the work or revising it critically for important intellectual content and made substantial contributions to the concept and design of the study and acquisition, analysis, and interpretation of data.

## Funding

This study was funded by the National Key R&D Program of China (2016YFC1307000), Capital health development research (2016-2-4112), Clinical Medicine Plus X - Young Scholars Project of Peking University (PKU2018LCXQ008), National Basic Research Program of China (973 Program, 2015CB856404), National Natural Science Foundation of China (91432304), and Natural Science Foundation of Shandong Province (ZR2019BH001), and Medical and Health Science and Technology Development Plan of Shandong Province (2018WS457).

## Conflict of Interest

The authors declare that the research was conducted in the absence of any commercial or financial relationships that could be construed as a potential conflict of interest.
