# Supplementary material for: Exploring the Causal Pathway From Telomere Length to Alzheimer’s Disease: An Update Mendelian Randomization Study
Source: Front Psychiatry. 2019 Nov 15;10:843. doi: 10.3389/fpsyt.2019.00843 (PMC6873744; doi:10.3389/fpsyt.2019.00843)
Supplement: Supplementary file 1 [file DataSheet_1.docx]

**Supplementary Figure 1. Comparison of the Mendelian Randomization (MR) study and observational studies of the association between telomere length and AD.** We compared our MR results with a meta-analysis of traditional observational studies of telomere length in AD (Forero et al., 2016) using Cochran Q statistic.

**
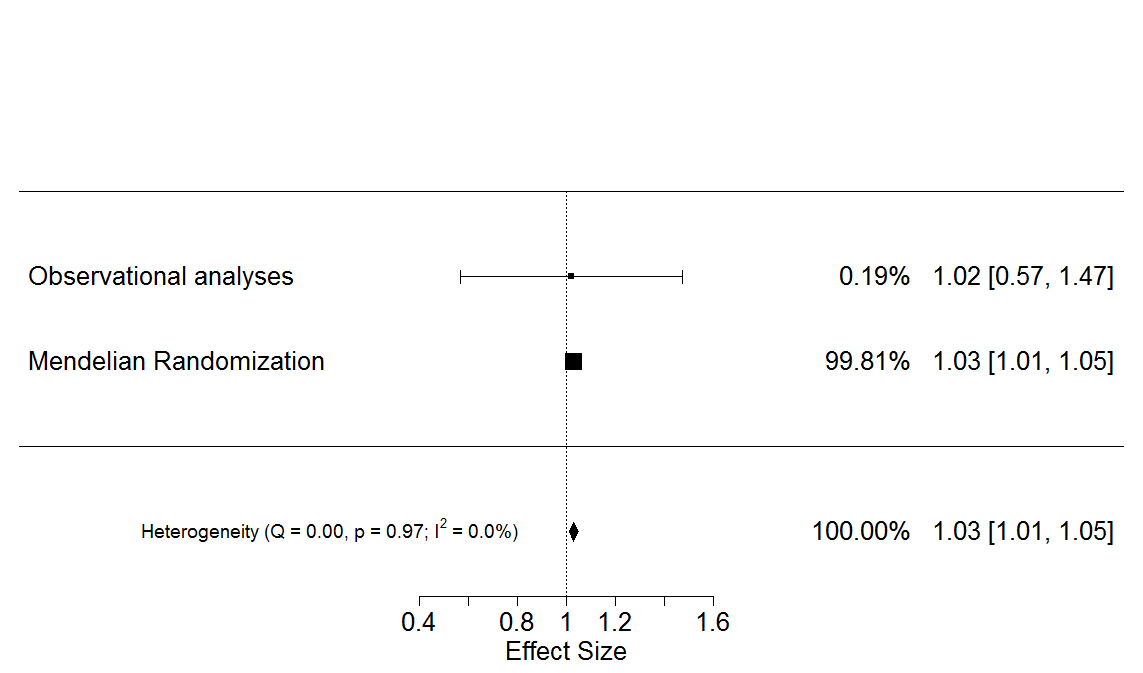
**

**Supplementary Table 1. Linkage disequilibrium-based clumping results of 16 genome-wide associated SNPs for telomere length.**

| **CHR** | **BP** | **SNP** | **P** | **Number** | **Clumped SNPs** |
| --- | --- | --- | --- | --- | --- |
| 3 | 169492101 | rs10936599 | 3.00E-31 | 3 | rs12696304,rs1317082,rs10936601 |
| 5 | 1286516 | rs2736100 | 4.38E-19 | 0 | N.A. |
| 4 | 164007820 | rs7675998 | 4.35E-16 | 0 | N.A. |
| 10 | 105677897 | rs4387287 | 2.00E-11 | 2 | rs9419958,rs9420907 |
| 3 | 58376019 | rs6772228 | 3.91E-10 | 0 | N.A. |
| 2 | 54475866 | rs11125529 | 8.00E-10 | 0 | N.A. |
| 19 | 22215441 | rs8105767 | 1.11E-09 | 1 | rs412658 |
| 20 | 62421622 | rs755017 | 6.71E-09 | 0 | N.A. |
| 17 | 8136092 | rs3027234 | 2.00E-08 | 0 | N.A. |
| 20 | 38129002 | rs6028466 | 2.57E-08 | 0 | N.A. |

Abbreviations: SNP, single-nucleotide polymorphism; Chr, chromosome; BP, base position; Number, the number of clumped SNPs; Clumped SNPs, the list of SNPs names clumped; N.A., not available.
